# Fibrolamellar Hepatocellular Carsinoma “It is not a Ordinary Case for Mediterranean Countries”

**DOI:** 10.1155/1995/63128

**Published:** 1995

**Authors:** Ismail Arslan, Köksal Öner, Sadik Kiliffturgay, Nihat Agar, Fahrettin Alpaslan

**Affiliations:** Gtilhane Military Medical Academy Ankara Turkey

## Abstract

The fibrolamellar variant of hepatocellular carcinoma (HCC) is an uncommon tumour with distinctive clinical and histological features. Although the first case of fibrolamellar hepatocellular carcinoma (FI-HCC) was described by Edmondson in 1956, and later confirmed in five patients by Peters, the majority of reports followed those of Berman *et al*. and Craig *et al*. ^1,2^.

A case of FI-HCC, which is rarely diagnosed in Mediterranean countries including Turkey, is presented in the following report.


*HPB Surgery*, 1995, Vol. 9, pp. 51–53
Reprints available directly from the publisher
Photocopying permitted by license only

© 1995 OPA (Overseas Publishers Association)
Amsterdam B.V. Published in The Netherlands
by Harwood Academic Publishers GmbH
Printed in Malaysia

CASE REPORT

### Fibrolamellar Hepatocellular Carsinoma "It is not a Ordinary Case for Mediterranean Countries"

ISMAIL ARSLAN, KÖKSAL ÖNER, SADIK KILIÇTURGAY,

NIHAT AGAR and FAHRETTIN ALPASLAN

Gülhane Military Medical Academy, Ankara

(*Received November 14, 1994*)

The fibrolamellar variant of hepatocellular carcinoma (HCC) is an uncommon tumour with distinctive clinical and histological features. Although the first case of fibrolamellar hepatocellular carcinoma (FI-HCC) was described by Edmondson in 1956, and later confirmed in five patients by Peters, the majority of reports followed those of Berman *et al.* and Craig *et al.*[1,2].

A case of FI-HCC, which is rarely diagnosed in Mediterranean countries including Turkey, is presented in the following report.

KEY WORDS: Hepatoma fibrolamellar carcinoma

### CASE REPORT

A 21-year-old male without a history of chronic alcohol abuse and cirrhosis presented in April 1989, with a one month history of right upper abdominal pain and a mass, this was diagnosed as a liver abccess and operated on in an urban hospital. A large mass in the right lobe of the liver was found and only a biopsy was performed. Histological examination revealed HCC. Almost one year later, while he was doing his military service, this patient was admitted to our hospital because of right upper abdominal pain and a mass. Physical examination was unremarkable except for hepatomegaly (liver edge 5 cm. below the right costal margin) and in particular there were no stigma of chronic liver disease. His liver function tests were normal: alkaline phosphatase (AP) 258 Ü/L, aspartate transaminase 34 Ü/L, bilirubin (total and direct) 0.4 and 0.1 g/L, with a normal albumin (3.3 g/l) and protrombin time (13 second). Viral serology, hepatitis B surface antigen (HBsAg) were all negative and tumor markers including AFP (3.2 ng/ml) and carcinoembrionic antigen (CEA) (3.1 ng/ml) were normal. Only ferritin was moderately elevated (320 ng/ml). Ultrasound revealed a hypoechoic mass. Abdominal computed tomography scan (ACTS) revealed a well-circumscribed, hypodense, large mass (13 × 10 × 11 cm.) with some necrotic areas, in the right lobe of the liver (Fig. 1). Since these findings and patient's history were not typical of HCC, biopsy specimens which were taken at the first operation were reevaluated at our hospital and the findings supported FI-HCC.

A right hepatic lobectomy and cholecystectomy were performed using the finger fracture method in May 1990. The gross specimen demonstrated a well-circumscribed, moderately firm, gray-white, 13 × 10 × 7 cm. lobulated mass with a fine central stellate fibrous scar and septa radiating to the periphery (Fig. 2). The remainder of the liver was noncirrhotic and grossly unremarkable. No metastasis or lymph nodes were identified at operation. Histological examination of the tumor revealed the characteristic appearance of FI-HCC, with large polygonal cells lying within a

*Address for correspondence*: Dr. Sadik Kiliçturgay, Uludag Üniversitesi Tip Fakültesi, Genel Cerrahi ABD Bursg/Turkey.

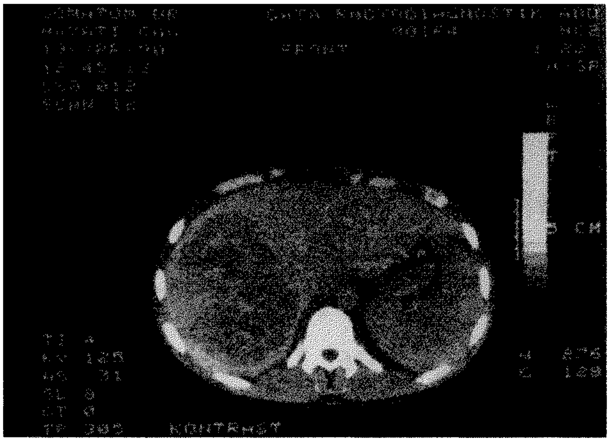

**Figure 1** CT scan showing a tumour mass in right site of the liver.

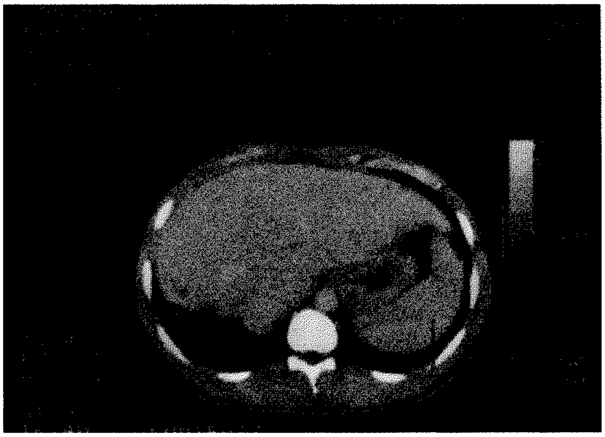

**Figure 4** Postoperative CT scan shows only hypertrophy of left side of the liver.

**Figure 2** Gross specimen demonstrates a lobulated mass with a white central stellate scar. See Color Plate I.

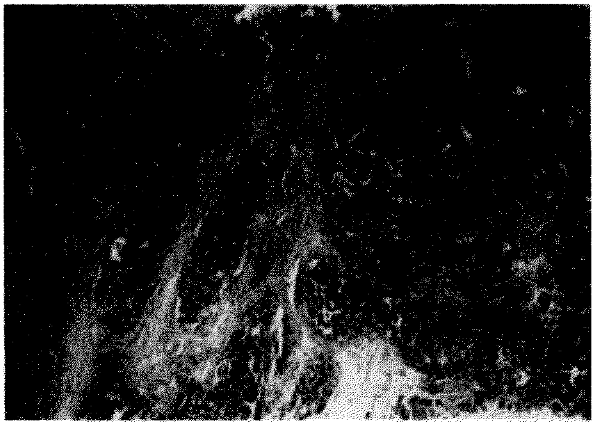

**Figure 3** Large polygonal cells are traversed by bands of fibrous tissue with a lamellar pattern (hemotoxylin and eosin stain × 100).

lamellar fibrous stroma (Fig. 3). The cells had eosinophillic cytoplasm, some with pale inclusions. With CEA stain CEA positive tumor cells were not found, only bile ducts showed a positive reaction. Follow up at 27 months failed to show recurrence of the tumor. In this last evaluation, ACTS revealed only hypertrophy of left lobe of liver (Fig. 4). Bone scintigraphy, routine biochemical and hematological values (including blood ferritin level) were all normal.

### DISCUSSION

HCC is a disease of widely varying epidemiology. It is relatively uncommon among adults in North America and Europe, where it is usually a disease of cirrhotic males aged 50 to 70 years. It is relatively more common in the Orient, Saudi Arabia and particularly in subsaharan Africa. Nevertheless, the majory of the FI-HCC have been reported from USA and especially there are very few cases reported from Mediterranean Countries[3].

The literature reveals that FI-HCC is a distinct clinical entity with a mean age of 23, less than 10 percent of the patients over 35 years of age and an almost equal sex ratio[4,5]. Patients with FI-HCC are more likely to have abdominal pain and a palpable abdominal mass or hepatomegaly. Sometimes, jaundice, phlebitis, hemoperitoneum or gynaecomastia may be seen[4]. By contrast with HCC, only 5 to 10 percent of patients with FI-HCC are positive for HBsAg and 10 to 14 percent have modest elevations of AFP levels. The diminished expression of AFP, an oncofetal antigen, in these tumors supports the concept of FI-HCC being a better differentiated tumor[4,5,6]. Serum unsaturated vitamin B12 binding capacity and plasma neurotensin level may be used to monitor the regression or recurrence of the tumor postoperatively[5]. Another reported parameter which is elevated in FI-HCC, is ferritin. However, ferritin has been reported to be elevated in not only cases of HCC but also some other neoplastic disease such as breast and lung cancers, neuroblastoma, multiple myeloma[5].

FI-HCC is usually a large, firm, grey solitary mass at the time of presentation, in contrast to HCC; which is often multiple, soft and hemorrhagic. FI-HCC frequently appears in a noncirrhotic liver as a well circumscribed mass with a central stellate scar and prominent fibrous tissue, reminiscent of focal nodular hyperplasia (FNH). A possible relationship of FI-HCC to FNH has been speculated[1,3]. Some authors have attempted to link FNH with FI-HCC suggesting that the latter may represent the malignant counterpart of the former[6]. This was based or their macroscopic similarities, their common age and gender, and the presence of FNH adjacent to FI-HCC in some of the early reports of FI-HCC. However, lots of studies revealed that only 5% of cases of FI-HCC had FNH identified in the same liver, providing little support for such an association.

The histologic criteria for FI-HCC are well defined and the diagnosis can be readily made by the identification of the characteristic large oncocytic tumor cells embedded within lamellar fibrosis. Since the first descriptions of FI-HCC, the presence of hyaline globules, focal ground glass cytoplasmic changes and intracytoplasmic "pale bodies" have been prominently described[1,2,4]. Our case is a typical sample of FI-HCC with this macroscopic and microscopic appearance.

The FI-HCC reported in the literature have a high resectabilty rate (48–100%)[1,2,3,4,5], where as, the resectabilty rate of patients with HCC is 10 to 23%[2,7]. The 5 year survival rate of the patients with FI-HCC who had complete tumor resection was 45 to 56%, with a median survival time of 50 to 68 months[1,3,5]. The 5 year survival of patients with HCC who had complete tumor resection was 0 percent, with median survival time 7–22 months[2]. The noncirrhotic fibrolamellar patients had significantly longer median survival than noncirrhotic hepatocellular carridone patients. Also fibrolamellar patients with solitary tumors survived twice as long as those with multiple lesions[7]. Although some authors have argued that patients with FI-HCC are ideal candidates for liver transplantation, resection appears to be an effective therapeutic modality[3,4,5,6].

In a conclusion, FI-HCC, seems to be a distinct clinical entity that mainly occurs in young patients. The prognosis in patients treated with a curative resection is good.

### REFERENCES

1. Bermah, M. M., Libbey, P. N. and Foster, J. H. (1980) Hepatocellular Carcinoma. Polygonal cell type with fibrous stroma. An atypical variant with a favorable prognosis. *Cancer*, **46**, 1448–55.
2. Craig, J. R., Peters, R. L., Edmondson, H. A. and Omata, M. (1980) Fibrolamellar carcinoma of the liver: A tumor of adolescents and young adults with distinctive clinicopathologic features. *Cancer*, **46**, 372–9.
3. Yoshida, K., Amemiya, A., Kobayashi, S., Sakurai, K., Suzuki, M. and Aizawa, S. (1988) Fibrolamellar carcinoma of the liver in the orient. *J. Surg. Oncol.*, **39**, 187–9.
4. Farhi, D. C., Shikes, R. H., Murari, P. J. and Silverberg, S. G. (1983) Hepatocellular carcinoma in young people. *Cancer*, **52**, 1516–25.
5. Soreide, O., Czemiak, A., Bradpiece, H., Bloom, S. and Blumgart, L. (1986) Characteristics of fibrolamellar hepatocellular carcinoma. A Study of nine cases and a Review of the literature. *Am. J. Surg.*, **151**, 518–23.
6. Berman, M. A., Burnham, J. A. and Sheahan, D. G. (1988) Fibrolamellar carcinoma of the liver: An immunohistochemical study of nineteen cases and a review of the literature. *Human Pathology*, **19**, 784–94.
7. Wood, W. J., Rawlings, M., Evans, H. and Lim, C. N. H. (1988) Hepatocellular Carcinoma: Importance of histologic classification as a prognostic factor. *Am. J. Surg.*, **155**, 663–6.

